# Electrophysiological responses to regularity show specificity to global form: The case of Glass patterns

**DOI:** 10.1111/ejn.14709

**Published:** 2020-03-09

**Authors:** Giulia Rampone, Alexis D. J. Makin

**Affiliations:** ^1^ School of Psychology University of Liverpool Liverpool UK; ^2^ Department of Psychology University of Liverpool Liverpool UK

**Keywords:** ERPs, Glass patterns, global representation, holographic model, perceptual goodness, visual symmetry

## Abstract

The holographic weight of evidence model (van der Helm & Leeuwenberg, *J Math Psychol*, 35, 1991, 151; van der Helm & Leeuwenberg, *Psychol Rev*, 103, 1996, 429) estimates that the *perceptual goodness* of moiré structures (Glass patterns), irrespective of their global form, is comparable to that of *reflection* symmetry. However, both behavioural and neuroscience evidences suggest that certain Glass forms (i.e. *circular* and *radial* structures) are perceptually more salient than others (i.e. *translation* structures) and may recruit different perceptual mechanisms. In this study, we tested whether brain responses for *circular*, *radial* and *translation* Glass patterns are comparable to the response for onefold bilateral *reflection* symmetry. We recorded an event‐related potential (ERP), called the sustained posterior negativity (SPN), which has been shown to index perceptual goodness of a range of regularities. We found that *circular* and *radial* Glass patterns generated a comparable SPN amplitude to onefold *reflection* symmetry (in line with the prediction of the holographic model), starting approx. 180 ms after stimulus onset. Conversely, the SPN response to *translation* Glass patterns had a longer latency (approx. 400 ms). These results show that Glass patterns are a special case of visual regularity, and *perceptual goodness* may not be fully explained by the holographic identities that constitute it. Specialised processing mechanisms might exist in the regularity‐sensitive extrastriate areas, which are tuned to global form configurations.

## Graphical abstract

In this study, EEG‐ERP responses (i.e. Sustained Posterior Negativity, SPN) for *circular* and *radial* Glass patterns were comparable to response to onefold *reflection* symmetry, whilst response to *translation* Glass patterns had longer latency. This suggests specialised processing mechanisms, tuned to global form configurations, might exist in the regularity‐sensitive extrastriate areas.

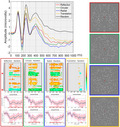

**Abbreviations**
Adj.: adjusted
ANOVA: Analysis of Variance
APA: American Psychological Association
approx.: approximately
CI: confidence intervals
CRT: cathode ray tube
e.g.: exempli gratia
EEG: electroencephalogram
ERP: event‐related potential
fMRI: functional magnetic resonance imaging
GFP: Global Field Power
HEOG: horizontal bipolar electrodes
i.e.: id est
ICA: independent components analysis
IQR: interquartile range
LOC: lateral occipital cortex
ms: milliseconds
obs.: observed
Pcorrect: proportion correct
Q1: first quartile
Q3: third quartile
RF: radial frequency
RTs: response times
*SD*
: standard deviation
SPN: sustained posterior negativity
TMS: transcranial magnetic stimulation
VEOG: vertical bipolar electrodes
W: weight of evidence (holographic model)
Δμ: difference mean
*η* ^2^
: eta‐squared
μV: microvolts

## 1 INTRODUCTION

Visual regularities are a special feature of images, which play a key role as cues for perceptual organisation. One key characteristic of regularity is its *perceptual goodness*, a Gestalt concept that relates to the notion of “Prägnanz” (Koffka, 1935; Köhler, 1929). Goodness refers to the perceptual *salience*, or *strength* of a given regularity, and can be empirically measured in terms of speed and accuracy of detection (Attneave, 1954; Barlow & Reeves, 1979; Bertamini, Friedenberg, & Kubovy, 1997; Koffka, 1935; Palmer, 1983)*.* In general, the greater the saliency of the regularity, the more efficient its visual processing. Despite this being an intuitive definition, the nature of the phenomenon remains challenging and not fully understood.

Several theoretical models have been proposed to describe the perceptual goodness of a regularity and predict the efficiency of processing (Garner, 1974; van der Helm & Leeuwenberg, 1991, 1996, 1999; Wagemans, Gool, Swinnen, & Horebeek, 1993). The Holographic Weight of Evidence model (van der Helm & Leeuwenberg, 1991, 1996)^1^ provides a simple framework to quantify *perceptual goodness* based on weight of evidence (*W*). The formula *W* = *E*/*N* takes into account the (*holographic*) *identities* that constitute a given regularity (*E*) and the total visual information within the regularity (*N*). In a simple way, *E* could be seen as the amount of redundant information; the more arrangements of *N* are repeated, the larger the *E*‐value. This model identifies a number of regularities with holographic properties, that is *mirror* (*reflection*) symmetry, *repetition* (*translation*) symmetry, *centric* (*rotation*) symmetry and Glass patterns.^2^ *W* can be easily calculated for dot patterns. For example, for a bilateral *reflection*, *N* is the number of dots, for example *N* = 10, and *E* is number of parallel dot pairs with a midpoint falling with the axis of *reflection*, for example *E* = 5. This means that *W* = 0.5, and *W* is unvaried with increasing number of dots (and pairs). The predictions of the holographic model have been tested empirically and have been shown to accurately relate to behavioural performance (Nucci & Wagemans, 2007) and neural activity (Makin et al., 2016).

Makin et al. (2016) conducted a large EEG/ERP study to investigate whether the *W*‐metric predicts the amplitude of the symmetry‐related component: the sustained posterior negativity (SPN; Bertamini, Silvanto, Norcia, Makin, & Wagemans, 2018; Höfel & Jacobsen, 2007; Jacobsen & Höfel, 2003; Jacobsen, Klein, & Löw, 2018; Makin, Wilton, Pecchinenda, & Bertamini, 2012; Martinovic, Jennings, Makin, Bertamini, & Angelescu, 2018; Wright, Mitchell, Dering, & Gheorghiu, 2018). Briefly, the SPN is a relative component given by the difference in amplitude between ERPs generated by symmetrical (regular) and asymmetrical (irregular) images with same local information. It has negative amplitude and is generated by neurons in the extrastriate cortex and lateral occipital complex (LOC; Makin, Pecchinenda, & Bertamini, 2012; Makin et al., 2016; Rampone, Makin, Tatlidil, & Bertamini, 2019). The SPN is a well‐characterised neural signal, and its interpretation is consistent with fMRI (Chen, Kao, & Tyler, 2007; Keefe et al., 2018; Kohler, Clarke, Yakovleva, Liu, & Norcia, 2016; Sasaki, Vanduffel, Knutsen, Tyler, & Tootell, 2005; Tyler et al., 2005; Van Meel, Baeck, Gillebert, Wagemans, & Op de Beeck, 2019) and TMS evidence (Bona, Cattaneo, & Silvanto, 2016; Bona, Herbert, Toneatto, Silvanto, & Cattaneo, 2014; Cattaneo et al., 2014; Cattaneo, Bona, & Silvanto, 2017). Makin et al. (2016) tested specific predictions of the holographic model. The *W*‐score predicted the SPN amplitude (i.e. greater *W*‐score larger SPN amplitude) remarkably well across a range of regularities.

**Figure 1:**
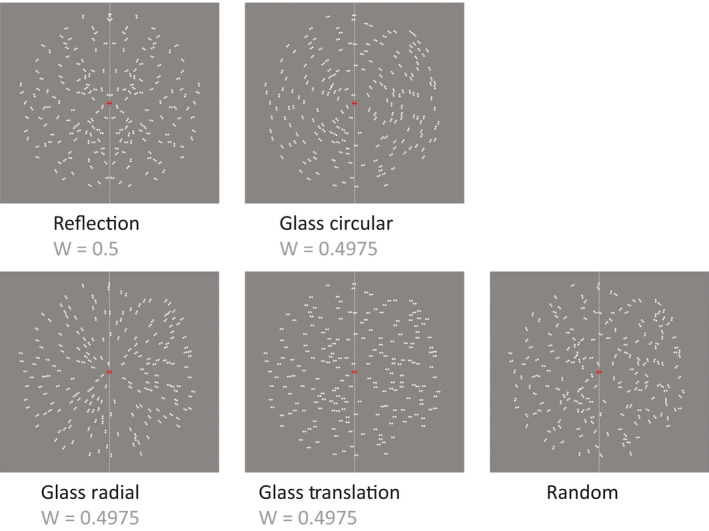
Examples of each pattern configuration used in the experiment. From left to right: reflection, circular Glass, radial Glass, translation Glass and Random pattern. Each pattern contained 200 dipoles in total (100 on each side of the central vertical meridian; this is indicated by the vertical line, which is shown here for illustration purposes). Dipoles positions coordinates were seeded, so that all patterns were equivalent in terms of dipoles distribution (in the figures, all patterns have same seed number); only the orientation of the dipoles differed according to the specific geometrical rules. Note that for reflection patterns, only the left half was comparable to the other patterns, as in this case the right half mirrored the left [Colour figure can be viewed at wileyonlinelibrary.com]

There is an important caveat in Makin et al. (2016), which concerns the similarity between Glass patterns and reflection symmetry. Glass patterns are moirés formed by identical dipoles (pairs of dots) that are randomly positioned but coherently oriented according to specific geometric transformations (Glass, 1969; Glass & Pérez, 1973). These patterns convey the percept of global forms thanks to the integration of orientation cues created by the locally paired dots. Several types of Glass global forms can be generated, that is *circular* (or *concentric*), *radial* and *translation* (or *parallel*; see Figure 1), whilst maintaining the same local stimulus statistics and same spatial frequencies across the different configurations.

van der Helm and Leeuwenberg (1996) suggest that Glass patterns have relevant holographic properties, such as *alternation*, which make them approximately as salient as onefold *reflection* symmetry. According to the holographic model, a moiré structure has a *W* = (*P* − 1)/(2*P*), with *P* = number of oriented dipoles. This gives a *W*‐score, which depends on increasing number of dipoles up to approximately a *W* = 0.5 asymptote (comparable to *W* for bilateral *reflection*). According to this formula, every type of Glass pattern has the same *W*‐score. Of course, this is not to say that the visual system treats reflection and different Glass patterns with the same *W*‐score as the same thing. The holographic model quantifies the perceptual goodness of various configurations, but does not explain why people can easily discriminate between different configurations of the same goodness.

In Makin et al. (2016)’s study 2, SPN amplitude was similar for onefold *reflection* symmetry and *circular* Glass patterns, confirming the prediction of the holographic model. Here, only *circular* Glass pattern was reported, as representatives for all forms of moiré structure, and the theoretical prediction is that all forms should generate a comparable SPN amplitude.

*Circular* Glass patterns are, however, a special case. Psychophysical studies have shown that the detection of *circular* or *radial* Glass patterns is easier than for *translation* Glass patterns (Kelly, Bischof, Wong‐Wylie, & Spetch, 2001; Kurki & Saarinen, 2004; Seu & Ferrera, 2001; Wilson & Wilkinson, 1998; Wilson, Wilkinson, & Asaad, 1997). This suggests the existence of specialised mechanisms that can discriminate among the different configurations. One theory is that fast detectors for global form located in higher extrastriate areas (e.g. V4) may be tuned to *circular* and *radial* Glass patterns, as well as complex stimuli such as polar and hyperbolic gratings; *translation* Glass patterns instead may require slower local‐to‐global integration processing (Achtman, Hess, & Wang, 2003; Anzai, Peng, & Van Essen, 2007; Clifford & Weston, 2005; Dumoulin & Hess, 2007; Gallant, Connor, Rakshit, Lewis, & Van Essen, 1996; Hegdé & Van Essen, 2007; Kelly et al., 2001; Kurki & Saarinen, 2004; Lestou, Lam, Humphreys, Kourtzi, & Humphreys, 2014; Li & Westheimer, 1997; Maloney, Mitchison, & Barlow, 1987; Ostwald, Lam, Li, & Kourtzi, 2008; Pavan, Hocketstaller, Contillo, & Greenlee, 2016; Pei, Pettet, Vladimir, & Norcia, 2005; Seu & Ferrera, 2001; Wilson & Wilkinson, 1998, 2015; Wilson et al., 1997; Wilson, Switkes, & De Valois, 2004).

Pei et al. (2005) recorded event‐related potentials (ERPs, over seven posterior electrodes placed at the level of Oz) in response to *circular*, *radial* and *translation* Glass patterns, versus random patterns with identical dipole local structure. Glass patterns were alternated with random patterns at 1 Hz (500 ms Glass pattern, 500 ms random). The onset of *circular* and *radial* patterns elicited more negative responses than the random control patterns from approx. 150 to 300 ms after stimulus onset, over posterior electrodes. Conversely, their offset elicited more positive responses at same latencies. Response to *translation* Glass pattern was not significantly different from response to random patterns. The authors tested these asymmetric response components in a second identical experiment (using a 128‐electrode array), which measured responses in the frequency domain. They observed odd harmonic activity for *circular* and *radial* patterns (indexing all aspects of the response that differentiates between the appearance and disappearance of the Glass pattern) and only even harmonic activity for *translation* patterns (corresponding to aspects of the waveforms arising from the image update, unrelated to the pattern's structure). Note that response to symmetry is also predominantly found in the odd harmonics (Kohler et al., 2016; Norcia, Candy, Pettet, Vildavski, & Tyler, 2002; Oka, Victor, Conte, & Yanagida, 2007).

This evidence contrasts with the predictions of the holographic model (van der Helm & Leeuwenberg, 1996) and the general conclusion of Makin et al. (2016). If all moiré structures have the same *holographic* properties, they should all be equally salient and generate the same SPN response. van der Helm and Leeuwenberg (1996) suggested that holographic account might not fully explain the perceptual aspects of different Glass patterns. It should be also acknowledged that other *representational models*, that is the *transformational model* (Garner, 1974), make different predictions regarding the perceptual impression evoked by Glass patterns. In the *transformational* account, circular Glass patterns are comparable to twofold centric (rotation) symmetry and translation Glass patterns are comparable to twofold repetition (translation) symmetry. These two different types of symmetry elicit SPN responses, which are less strong than SPN elicited by twofold reflection symmetry (Makin, Rampone, Pecchinenda, & Bertamini, 2013). Makin et al. (2016), however, showed that *circular* Glass patterns elicited same SPN as *reflection* symmetry as predicted by the holographic model.

In summary, Makin et al. (2016) tested the relation between pattern's *holographic* properties and brain responses only with *circular* Glass patterns. This leaves open the question of whether the *W* would explain variance in SPN for other Glass patterns, such as *radial* and *translation*.

In this study, we recorded ERPs responses to three different types of Glass patterns, as well as bilateral *reflection* symmetry, and contrasted these responses to those obtained from random patterns with identical local dipolar structure. The patterns were presented for 1,500 ms, and participants classified them as either regular or random. In addition, we conducted a separate behavioural detection task experiment where participants had to discriminate the same patterns (as regular or random) as quickly and accurately as possible. This was meant to assess consistency across different measures of perceptual goodness (i.e. W, SPN, RTs and error rates). Makin et al. (2016) observed that *W*‐score predicted the amplitude of SPN as well as RTs and error rates, and all these measures were strongly correlated (see also Makin, Helmy, & Bertamini, 2017; Nucci & Wagemans, 2007).

For simplicity, we did not include a *translation* symmetry condition. This was tested in study 2 of Makin et al. (2016), who observed that a onefold *translation* symmetry pattern (*W* = 0.01) produces an equivalent ERP response to a random control pattern. Here, we limited the focus on pattern conditions with comparable *W*‐score.

## 2 ERP STUDY

### 2.1 Method

#### 2.1.1 Participants

Twenty‐four participants took part in the experiment (age 18–23, mean 19.4, *SD* 1.2, males: 9, left handed: 4). Participants had normal or corrected‐to‐normal vision (i.e. through lenses); participants’ suitability to perform the task was established by assessing their ability to discriminate the shapes correctly during the practice section. Some received either course credit or financial reimbursement upon completion of the study. The study was approved by the University of Liverpool Ethics Committee (ethics reference number: 2122) and conducted in accordance with American Psychological Association (APA) code of practice (2017). The experiment was conducted largely in accordance with the Declaration of Helsinki (although the study was not pre‐registered, which is required by point 35 of the 2008 revision).

#### 2.1.2 EEG apparatus

EEG activity was recorded using a BioSemi Active‐Two amplifier in an electrically shielded and darkened room. EEG data were sampled continuously at 512 Hz from 64 scalp electrodes embedded in an elasticised cap arranged according to the standard international 10–20 system. In order to detect blinks and eye movements online, vertical bipolar electrodes (VEOG) were positioned above and below the right eye. Horizontal bipolar electrodes (HEOG) were positioned on the outer canthi of both eyes. Stimuli and experiment were programmed using the PsychoPy software (Peirce, 2007) and presented on a CRT monitor (60 Hz refresh rate; resolution: 1,280 × 1,024). Participants were positioned 100 cm from the monitor with their head stabilised in a chin rest. The same apparatus was used in Makin et al. (2016).

#### 2.1.3 Stimuli

Five different pattern configurations were used in the experiment: three Glass patterns (concentric, *radial* and *translation*), a random pattern and a onefold *reflection* symmetry pattern. All patterns consisted of white dot dipoles (RGB [1,1,1], luminance 168.5 cd/m^2^) presented on a grey background (RGB [0,0,0], luminance 30.2 cd/m^2^; RGB colour space is expressed as deviations from grey ranging between −1 and 1; Peirce, 2007). All patterns in this experiment were generated afresh and were different from each other; no participant ever saw the same pattern twice.

Dipoles were made with two dots with radius 0.04°. The radius of a dipole (distance from the centre of a dot and the centre of the dipole) was 0.08°. Dipoles locations were restricted within the circumference of an outer *circular* region with radius 6.4° and an inner *circular* region, around the central fixation point, with radius 0.5°. Minimum distance between dipoles was 0.26°. Figure 1 shows an example of each pattern. All patterns were made of 200 dipoles (100 dipoles on each side of the central vertical meridian). *W*‐score for Glass patterns was *W *= 199/400 = 0.4975, whilst *W *for reflection was *W* = 100/200 = 0.5.

For each pattern, a list of coordinates, corresponding to the position of the centre of each dipole, was generated afresh at the beginning of each trial. This process was controlled by applying a *seed* number to the random generation of the dipoles coordinates. For all patterns, the same *seed* number list (1–60, plus additional 60–240 for random patterns) was used. This process allowed to create identical copies of the same pattern which only differed for the orientation of the dipoles. In other words, for each random pattern s_1,_ there was a corresponding *circular* s_1_, *radial* s_1_ and *translation* s_1_ pattern. In these patterns, the dipoles position within the large *circular* region was the same. The only difference was the orientation of the dipoles according to the specific geometrical rule. *Reflection* patterns were also *seeded*. In this case, dipoles positions in the left half of the *reflection* pattern s_1_ were same as dipoles positions in the left half of the other corresponding patterns s_1_. This approach was used to minimise any difference in local statistics between the patterns.

**Figure 2:**
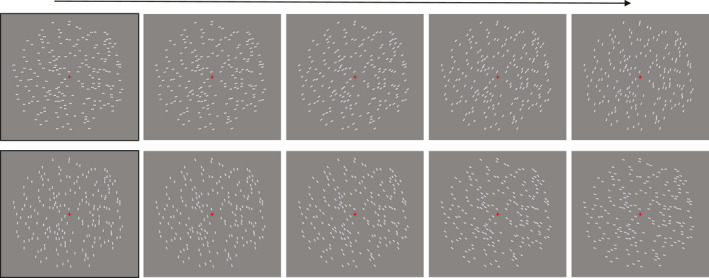
For translation Glass patterns, there were 10 different variations. These depended on the orientation of the dipoles, which ranged between 0° and 180° in steps of 18° (in the figures all patterns have same seed number) [Colour figure can be viewed at wileyonlinelibrary.com]

The orientation of the individual dipoles depended on a specific geometric rule for each type of pattern configuration (Glass, 1969; Glass & Pérez, 1973). For the *radial* Glass patterns, the angle for each dipole was formed by the *x*‐axis of the Cartesian plane and a vector starting from the origin (0,0) and terminating at the coordinates (*x*, *y*) of the centre of the dipole. In this way, dipoles were oriented orthogonally to the circumference of the *circular* region centred at fixation. In the *circular* Glass patterns, the dipoles were positioned tangentially to the circumference. For the *translation* Glass patterns, the orientation of the dipoles ranged between 0° and 180° in steps of 18° resulting in 10 different stimuli for each of the 10 axis orientations. In a random pattern, a dipole could get any orientation angle on a range between 0° and 180°. For *reflection* patterns, the orientation of the dipoles was assigned randomly for the left half of the pattern (in the same way as for the random patterns). Dipoles positions and orientations on the right‐half side of the pattern mirrored the left‐half side (Figure 2).

#### 2.1.4 Procedure

Prior to the beginning of the experiment, participants completed a practice block. This consisted of 28 trials, with two examples for each stimulus conditions (including two exemplars for each orientation of the *translation* pattern). The real experiment consisted of a total 480 trials (60 repetitions for each regular stimulus and 240 repetitions for the random stimulus). Participants were asked to fixate centrally throughout the baseline (1,500 ms) and stimulus presentation (1,500 ms). They were allowed to have a rest and break fixation during the experiment, which was divided into 16 blocks. The response task consisted on discriminating Regular (i.e. *reflection*, *circular* Glass, *radial* Glass, *translation* Glass) from Random patterns, after the stimulus disappeared. Participants entered a response by pressing either the “A” or “L” button of the computer keyboard with their left or right index fingers. A response screen was presented indicating how to respond (i.e. “Regular Random” or “Random Regular,” counterbalanced across trials). Responses were required to be as accurate as possible, whilst response speed was not measured. This was intended to minimise motor responses during the stimulus presentation period.

#### 2.1.5 EEG analysis

EEG data were processed using the EEGLAB toolbox in MATLAB (Delorme & Makeig, 2004). Pre‐processing conventions followed our previous studies (Makin et al., 2016). Data were referenced to a scalp average and down‐sampled to 128 Hz. We then segmented the data into −1‐ to 2‐s epochs. Independent components analysis (ICA) was used to remove oculomotor and other gross artefacts. On average, 9.4 (*SD* = 3.5) out of 64 components were removed from each participant (min = 3, max = 18). After ICA, trials, where amplitude exceeds ± 100 μV at any electrode, were excluded. Trials, where participants entered incorrect response, were still included in ERP analysis (mean response error rate ranged between 3% and 5%, and exclusion of the error trials from average ERPs did not change the results). After application of these exclusion criteria, the grand‐average ERPs were calculated on over 90% of the original trials for each condition.

**Figure 3:**
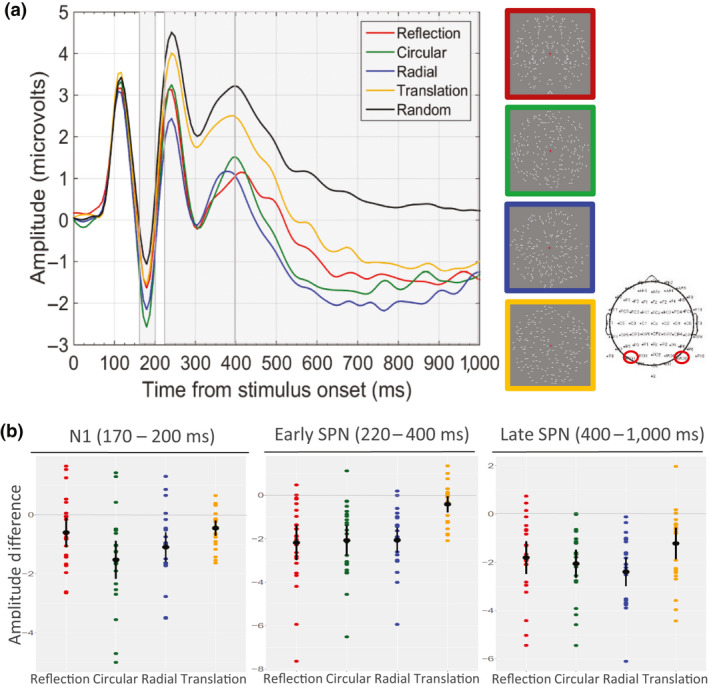
(a) Grand‐average ERPs calculated from activity over posterior electrodes PO7 and PO8 (the head cap depicts the Biosemi 64‐channel layout). The frame surrounding each exemplar indicates the colour of the corresponding ERP wave (red = reflection, green = circular Glass, blue = radial Glass, yellow = translation Glass). Dark‐grey shades indicate the three time windows analysed: N1 (170–200 ms), Early SPN (220–400 ms), Late SPN (420–1,000 ms). (b) Stripcharts (i.e. one‐dimensional scatter‐dot plots) showing distributions of individual participants’ difference amplitudes for each condition (reflection, circular Glass, radial Glass, translation Glass)—random, at the three time windows analysed. Mean difference amplitude is superimposed (black dot), and error bars indicate 95% confidence intervals [Colour figure can be viewed at wileyonlinelibrary.com]

**Figure 4:**
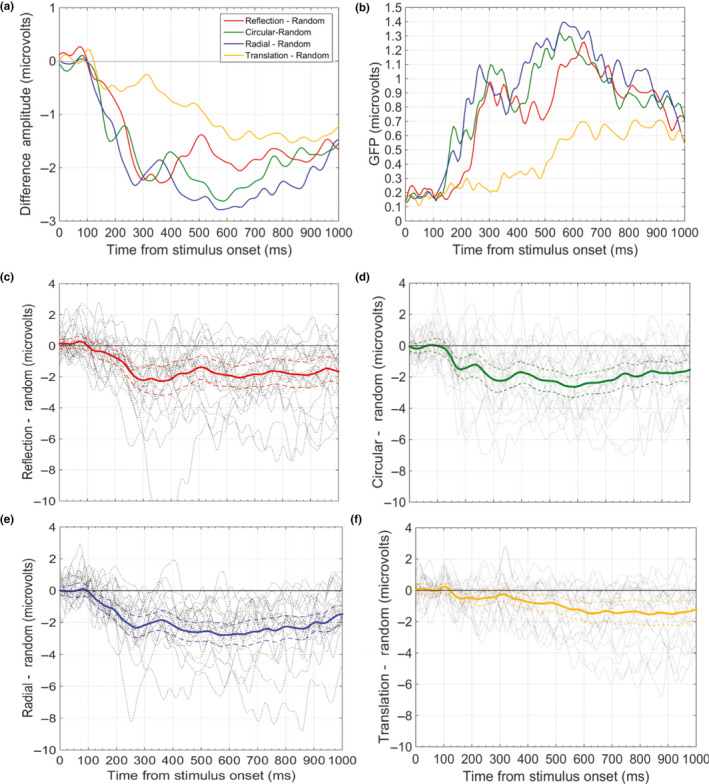
(a) Grand‐average ERP difference waves for each pattern configuration—Random calculated from activity over posterior electrodes PO7 and PO8. (b) Global Field Power (GFP) plot showing standard deviation of amplitude, for each difference wave, across the 64 electrodes. (c) Difference wave for the reflection − random contrast, plotted with 95% CI (dotted waves) and individual participant traces in the background (grey). Difference wave with 95% CI and individual traces for the circular Glass − random contrast (d), for the radial Glass − random contrast (e) and for the translation Glass − random contrast (f). When CI are below zero, the difference wave is significant at the .05 level (note that for translation Glass this happens from approx. 400 ms) [Colour figure can be viewed at wileyonlinelibrary.com]

**Figure 5:**
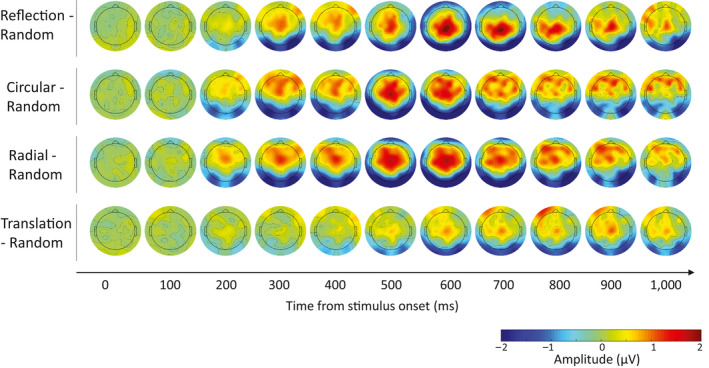
Topographic maps of the difference ERP for each Regular (reflection, circular Glass, radial Glass, translation Glass)—Random pattern. Each map shows average scalp activity every 40 ms starting from stimulus onset to end of the epoch. The amplitude range is ± 2 μV (negative amplitudes in dark blue and positive amplitudes in dark red) [Colour figure can be viewed at wileyonlinelibrary.com]

We were interested in testing the activation of the extrastriate symmetry network for Glass patters; mean ERPs at posterior PO7 and PO8 electrodes were computed (see Figure 3), based on previous ERP studies on similar patterns (Makin et al., 2016). Grand‐average N1 amplitude was calculated between 170 and 200 ms after stimulus onset. Both ERP plots in Figures 3a and 4, and topographic maps in Figure 5, show that individual SPNs change across the epoch (i.e. the latency of the response to *translation* Glass is delayed to approx. around 400 ms). We thus broke the SPN into two separate time windows: 220–400 ms (early SPN) and 400–1,000 ms (late SPN). The decision to consider an early and late SPN separately is justified by recent research (Makin et al., 2016; Rampone et al., 2019; Wright, Makin, & Bertamini, 2017). In the early time window, amplitude has been found to maximally correlate with a quantitative index of perceptual goodness. The strength of the correlation declines after the early peak. None of the five ERP conditions deviated significantly from normality according to the Shapiro–Wilk test (*p *> .05), in any of the components and time windows analysed. Data were thus analysed with repeated measures ANOVA.^3^ The Greenhouse–Geisser test correction factor was applied when the assumption of sphericity was violated.

In addition to ERP amplitudes, we also computed Global Field Power (GFP). GFP is the standard deviation of amplitude across the 64 electrodes at a particular time point (and quantifies the degree of the colour‐variation in a topographic map). GFP takes all the electrodes into account; therefore, it can be used as further evidence that results are not dependent on electrode choice.

**Figure 6:**
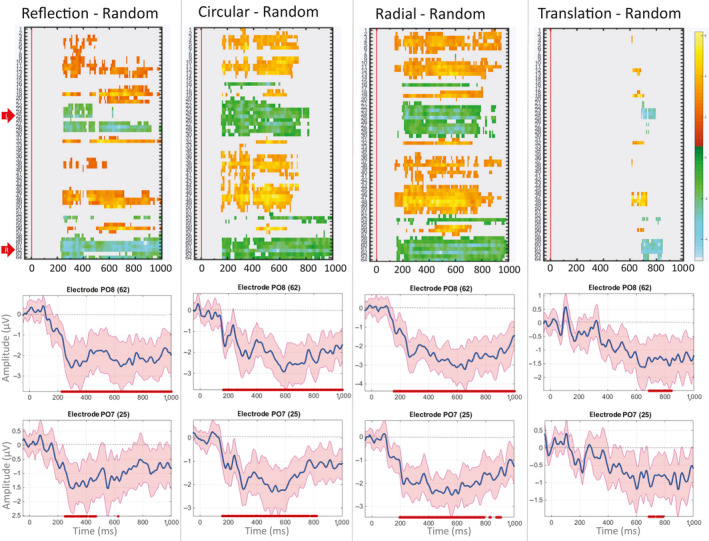
Mass univariate analysis. Top panels show results from multilevel pairwise comparisons (from left to right: reflection—random, circular Glass—random, radial Glass—random, translation Glass—random). Colour scale represents *t*‐values (positive values are in yellow/orange; negative values in blue/green). The comparisons are corrected by spatiotemporal cluster‐based computational methods. All tests where *p *> .05 appears grey. *X*‐axis shows time from stimulus onset (indicated by red line; baseline is −50 ms); *Y*‐axis shows electrode number from the BioSemi 64 electrode montage. Red arrows indicate L = left and R = right electrodes selected a priori for the ERP analysis (PO7/PO8). Middle and bottom rows show pairwise comparison from the selected individual electrodes with correction by spatial–temporal cluster (shaded red area indicates 95% confidence intervals; red dots below indicate significant difference from zero at *p* < .05 level) [Colour figure can be viewed at wileyonlinelibrary.com]

We have further assessed the spatiotemporal development of the regularity response by using mass univariate analysis, which computes a multilevel pairwise comparison at each electrode and time point. This was conducted using the hierarchical linear modelling toolbox for EEG (LIMO) MATLAB toolbox (Pernet, Chauveau, Gaspar, & Rousselet, 2011; Pernet, Latinus, Nichols, & Rousselet, 2015). The analysis deals both with within‐subject variance (i.e. single trial analyses) and between‐subject variance; data are analysed using a hierarchical general linear model where parameters are estimated for each subject at each time point and each electrode independently (1st level analyses). Estimated parameters from the first level analyses are then integrated across subjects (2nd level analysis). With this approach, we conducted pairwise comparisons (categorical variables: *reflection* vs. *random*; *circular* Glass vs. *random*; *radial* Glass vs. *random*; *translation* Glass vs. *random*) and applied spatiotemporal clustering for multiple comparisons correction. This is a rigorous correction method, which uses the distribution of bootstrap clusters defined simultaneously in space and time. An observed spatial–temporal cluster of *t*‐values is statistically significant if the sum of *t*‐values contained in the cluster is bigger than the threshold bootstrap cluster sum obtained under H0 (Pernet et al., 2011, 2015). Results are reported in Figure 6; a criterion *p*‐value of .05 was used; and all areas in grey correspond to *p *> .05.

### 2.2 Results

#### 2.2.1 Behavioural

Proportion correct (Pcorrect) differed between conditions, mainly because participants responded less accurately for *translation* Glass, χ^2^(3) = 9.0, *p* = .03). Median Pcorrect results were *reflection* 96.7% (range: 18%); *circular* Glass 96.7% (48%); *radial* Glass 96.7% (55%) and *translation* Glass 88.3% (50%).

#### 2.2.2 ERPs

Grand‐average ERPs from PO7/8 and difference waves are shown in Figures 3 and 4, respectively. There were some differences in N1 amplitude, with largest N1 for *circular* Glass and *radial* Glass. All regular patterns generated a SPN. The latency of the SPN for *translation* Glass was delayed compared with the other regularities (Figures 3, 4, 5). This is clearly shown in Figure 4a,c–f, where difference waves are plotted with 95% confidence intervals (CI; when CI cross zero, the difference wave is significant at the .05 level). For *translation* Glass, CI only cross zero at approx. 400 ms. For *circular* and *radial* Glass, CI cross zero at approx. 170 ms and just after 200 ms for *reflection*. GFP development over time (Figure 4b) parallels development of the SPN difference waves.

##### N1 (170–200 ms)

The main effect of regularity on N1 amplitude was significant, *F*_(2.92, 67.29)_ = 9.57, *p* < .001, partial *η* ^2^ = .3. Pairwise comparison (Bonferroni corrected) showed *circular* Glass’ N1 was significantly more negative than Random (Δμ = −1.5, *p* = .001, 95% CI = −2.5, −0.55), *reflection* (Δμ = −0.92, *p* = .05, 95% CI = −1.8, −0.001) and *translation* Glass (Δμ = −1.1, *p* = .03, 95% CI = −2.1, −0.08), whilst its difference from *radial* Glass was not significant (*p* = 1). N1 for *radial* Glass also significantly differed from Random (Δμ = −1.1, *p *= .002, 95% CI = −1.8, −0.34). No other significant differences were observed (*p*s > .05).

##### SPN

We conducted a 5 × 2 ANOVA with Regularity × Time window as factors. There was a main effect of Regularity, *F*_(4,92)_ = 24.9, *p* < .001, partial *η* ^2^ = .52 and a main effect of Time window, *F*_(1,23)_ = 25.6, *p* < .001, partial η^2^ = .53. Interestingly, the interaction Regularity × Time window was significant, *F*_(4,92)_ = 6.7, *p* < .001, partial *η* ^2^ = .23. Follow‐up tests of simple effects provided pairwise comparison of levels of Regularity, for each level of Time window (Bonferroni adjusted). *Early SPN (220–400 ms):* differences from Random were observed for *circular* Glass (Δμ = −2.08, *p* < .001, 95% CI = −3.1, −1.04), *radial* Glass (Δμ = −2.06, *p* < .001, 95% CI = −2.9, −1.2) and *reflection* (Δμ = −2.18, *p* < .001, 95% CI = −3.4, −0.97). Amplitude for *translation* Glass was not significantly different from Random (Δμ = −0.41, *p* = .2, 95% CI = −0.97, 0.14). Translation Glass also differed from *circular* Glass (Δμ = −1.7, *p* = .001, 95% CI = −2.7, −0.62), *radial* Glass (Δμ = −1.6, *p* = .001, 95% CI = −2.5, −0.83) and *reflection* (Δμ = −1.8, *p* = .001, 95% CI = −2.9, −0.64). *Late SPN (400–1,000 ms):* amplitudes for all Regular patterns differed significantly from Random (*circular* Glass (Δμ = −2.06, *p* < .001, 95% CI = −2.9, −1.2), *radial* Glass (Δμ = −2.4, *p* < .001, 95% CI = −3.3, −1.5), *reflection* (Δμ = −1.8, *p* < .001, 95% CI = −2.8, −0.68) and *translation* Glass (Δμ = −1.3, *p* = .002, 95% CI = −2.2, −0.36)). Only *radial* Glass showed a significant difference from *translation* Glass (Δμ = −1.1, *p* = .003, 95% CI = −1.9, −0.3). No other significant differences were observed (*p*s > .1).

These results are also shown in the topographic maps of the Grand‐average ERPs at different time points (every 100 ms) along the epoch. *Circular* and *radial* Glass showed earlier latency (N1 level), whilst activity for *translation* Glass started later in the epoch.

#### 2.2.3 Mass univariate analysis

Mass univariate analysis confirmed the above ERP analysis. In the upper panels of Figure 6, time points and electrodes with significant differences from random are coloured. This provides a clearer illustration of the spatiotemporal development of significant SPN effects for each regularity condition. Again, there was a weaker and delayed SPN for *translation* Glass patterns compared with the other regularities (Figure 6), and early N1 latency response to *circular* and *radial* Glass. Indeed, with the more conservative spatiotemporal thresholding used here, *translation* Glass patterns only diverge from random for a brief interval beginning ~700 ms. These temporal dynamics are also shown in the lower panels of Figure 6, which illustrates the difference waves at the electrodes PO7 and PO8, and time points when amplitude is significantly < 0 (after correction).

## 3 BEHAVIOURAL STUDY

### 3.1 Method

#### 3.1.1 Participants

A separate group of 24 participants took part in the behavioural study (mean age 21.7, *SD* = 4.8; all females, one left handed). These were first year psychology students at University of Liverpool, who received course credits for their participation.

#### 3.1.2 Stimuli, procedure and design

Stimuli were same as those used in the EEG experiment and same number of trials (480 trials; 60 repetitions for each regular stimulus and 240 repetitions for the random stimulus). Participants sat 57 cm from the screen and their head movements were restricted by the use of a chin rest.

In this experiment, participants were required to discriminate between regular and random patterns as quickly and accurately as possible. Half trials required a “regular” response, and half trials required a “random” response. Participants pressed always the same button (either A or L) for one specific category (the order of the button was counterbalanced across participants). After a baseline interval of random duration (between 1 and 1.5 s), patterns were displayed at the centre of the screen for a maximum of 2 s. Participants were required to enter their response within this timeframe; otherwise, the response would be considered as a miss and marked as incorrect.

Shapiro–Wilk normality tests were conducted separately for each condition on RTs data (all significant, *p*s < .001) and error rates (all significant, *p*s < .1, except for translation Glass, *W* = 0.93, *p*‐value = .11). Data were thus analysed with non‐parametric Friedman's ANOVA (4 levels: reflection, circular, radial and translation). For response time (RT), we removed trials where participants pressed the incorrect button or missed response and trials where responses were faster than 150 ms. Errors rates included incorrect button press and missed responses (>2 s).

### 3.2 Results

**Figure 7:**
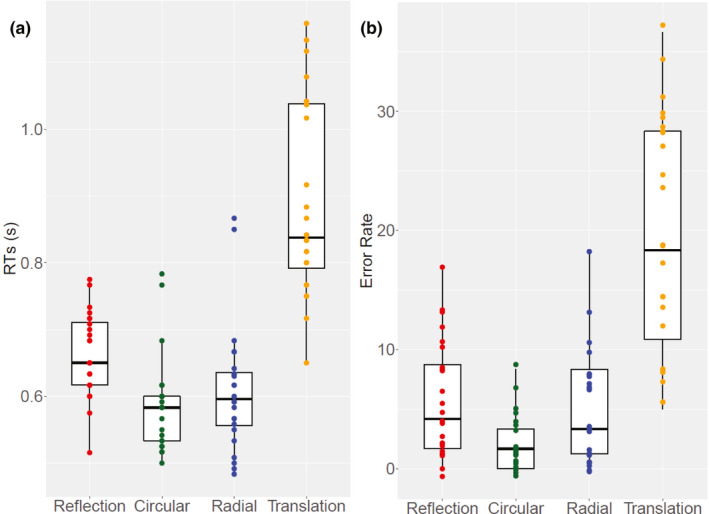
(a) Stripcharts (i.e. one‐dimensional scatter‐dot plots) showing the distribution of individual median response time (RT) on all correct‐response trials for each condition. (b) Stripcharts showing the distribution of individual proportion incorrect responses for each condition. Box plots are superimposed to provide descriptive statistics information. The dark black line in the middle of the box (in yellow) represents the median. The box represents the interquartile range (IQR) and extends from the first quartile (Q1) to the third quartile (Q3). The whiskers show values within 1.5 × IQR from the Q1 and Q3 [Colour figure can be viewed at wileyonlinelibrary.com]

Stripcharts (i.e. one‐dimensional scatter‐dot plots) showing individual median RTs distribution for each condition are shown in Figure 7a. Box plot is superimposed to provide interquartile range with median (dark line). RTs for *translation* Glass were slower than for the other conditions (and spread over a wider range of values, suggesting larger individual differences in participants’ discrimination speed for these structures). Values for the other regular patters were confined in a more limited range of faster RTs.

There was a significant difference in RTs between the four conditions χ^2^(3) = 54.35, *p* < .001. Post hoc multiple comparisons between groups were used, with Bonferroni correction applied. Critical difference for all cases (*α* = .05 corrected for number of tests) was 23.6. Median RTs for *reflection* were significantly slower than both *circular* Glass (observed difference = 31 ms) and *radial* Glass (obs. difference = 25 ms) patterns. *Circular* versus *radial* Glass were not significantly different (obs. difference = 6 ms). RTs for *translation* differed significantly from RTs for all the other patterns (difference *reflection* = 28; *circular* = 59; *radial* = 53).

Error rate results were very similar (Figure 7b). There were larger individual differences in participants’ ability to correctly identify *translation* Glass patterns as regular. There was greater consistency for the other patterns, with *circular* Glass leading to most correct identifications. There was significant difference across conditions χ^2^(3) = 53.382, *p* < .001. Post hoc multiple comparisons (Bonferroni corrected) showed significant difference *reflection* versus *circular* Glass (obs. difference = 24.5) but not *reflection* versus *radial* Glass (obs. difference = 13). C*ircular* versus *radial* Glass were not significantly different (obs. difference = 13). *Translation* differed significantly from all the other patterns (difference *reflection* = 36; *circular* = 60.5; *radial* = 47.5). Critical difference (*α* = .05 corrected for number of tests) was 23.6 for all cases.

## 4 DISCUSSION

We found comparable SPN responses to *circular* Glass patterns, *radial* Glass patterns and onefold vertical *reflection.* Conversely, the response for *translation* Glass patterns was weaker and only started diverging from *random* late in the epoch, approx. 400 ms after stimulus onset (although mass univariate analysis, with conservative spatiotemporal thresholds correction applied, only identified a response to *translational* Glass approx. 700–820 ms at posterior electrodes). Furthermore, participants were less likely to classify *translation* Glass patterns correctly. Behavioural data from the speeded‐detection task (experiment 2) showed similar results: participants generally had slower RTs for *translation* Glass patterns and made higher proportion of errors for this condition.

Our results partially contradict the holographic model, which predicts a similar SPN amplitude for all Glass pattern types and *reflection*, because they have similar W‐scores (van der Helm & Leeuwenberg, 1991, 1996; Makin et al., 2016). *Translation* Glass patters represent a special class, where perceptual goodness is not determined by the ratio of holographic identities to total information. Perhaps *translation* glass patterns are processed like translational symmetry, which also produces a much weaker SPN than *reflection* or *circular* Glass (Makin et al., 2016). This would be also partially in line with the predictions of the transformational model, which compares translation Glass patterns to repetition (translation) symmetry. This is a possibility, although translational symmetry with this number of elements would produce no SPN, even in the late window. Moreover, our results do support the holographic model by showing similar SPN waves for *reflection*, *circular* and *radial* Glass patterns.

Our results are in line with previous work that suggests different perceptual processes for different Glass pattern types (Achtman et al., 2003; Clifford & Weston, 2005; Dumoulin & Hess, 2007; Kelly et al., 2001; Kurki & Saarinen, 2004; Lestou et al., 2014; Li & Westheimer, 1997; Maloney et al., 1987; Ostwald et al., 2008; Pavan et al., 2016; Pei et al., 2005; Seu & Ferrera, 2001; Wilson & Wilkinson, 1998, 2015; Wilson et al., 1997, 2004). Detection of *radial* and *circular* Glass patterns may involve specialised configural pooling mechanisms, which include *linear* filtering of local orientation information (V1) followed by a full‐wave rectification, local pooling by larger second‐stage filters (V2), and finally global *linear* pooling by neurons in V4. Conversely, *translation* Glass patterns may follow a slower local‐to‐global integration process (Dobbins, Zucker, & Cynader, 1987; Wilson & Wilkinson, 1998, 2015; Wilson et al., 1997). These models are supported by single‐cell recordings from macaque V4, where neurons responded most to either *circular*, *radial*, *spiral* or *hyperbolic* gratings (Gallant, Braun, & Van Essen, 1993; Gallant et al., 1996; Kobatake & Tanaka, 1994). More recent psychophysical (Kelly et al., 2001; Kurki & Saarinen, 2004; Seu & Ferrera, 2001) and neuroscientific results (Ostwald et al., 2008; Pei et al., 2005) corroborate this theory.

Functional MRI in monkeys and humans has identified symmetry‐related activations in areas V4 and LOC but not areas V1 or V2 (Chen et al., 2007; Keefe et al., 2018; Kohler et al., 2016; Sasaki et al., 2005; Tyler et al., 2005; Van Meel et al., 2019). Van Meel et al. (2019) suggest a gradual change from part‐base coding in areas V1–V2, to computation of more complex features in V4, to final global symmetry representation in LOC. This is in line with the accounts of *circular* Glass pattern detection, where the system moves from local orientation tuning in V1 to curvature in V2 to closed curved shapes extraction in V4 (Wilson & Wilkinson, 2015).

Based on these observations, a speculative hypothesis might be that regularity‐specific processing may start in V4, where special units encode specific structures by pooling information from earlier visual filters. Gallant and colleagues defined some of these structures *non‐Cartesian* (i.e. *circular*, *radial*, *spiral* and *hyperbolic*, Gallant et al., 1993; Gallant et al., 1996; Kobatake & Tanaka, 1994), and it is interesting to note that some of these structures contain both *reflection* (i.e. cross‐shaped, hyperbolic) and *rotation* (i.e. spiral) symmetry. The following step of the hierarchy would then include weighting and pooling of a population code of V4 units into a final global representation at a higher cortical level, that is the LOC.

However, it is important to mention other studies that challenge the model originally proposed by Wilson et al. (1997) and Wilson and Wilkinson (1998). For instance, Dakin and Bex (2002) showed that the superiority for *circular* Glass patterns was only evident if the stimuli were presented in a circular window, which boundaries would provide significant perceptual advantage to the stimulus. No superiority for *circular* structure over *translation* was observed when patterns were presented through a square aperture or a round aperture surrounded by noise dots. We believe this may not be an issue with our stimuli, since all patterns were confined in a circular region and no dipole would be allowed to fall‐off the edge of the circular boundaries. *Radial* Glass and *reflection* structures should encounter the same disadvantage as *translation* Glass structures; however, their brain responses were more comparable to *circular* Glass than to *translation* Glass patterns. Schmidtmann, Jennings, Bell, and Kingdom (2015) measured summation for a variety of orientation‐defined textures, including Glass patterns (i.e. *circular*, *radial, spiral* or *translation)*, and found threshold detection sensitivities to be largely independent of patterns’ texture. Their results thus do not support the idea of specialised detectors for (circular) structures, as suggested by the models discussed above. Moreover, the authors reject the aforementioned *linear (additive) summation hypothesis* (i.e. linear summation of the local information signals into global form representation) and propose a *probability summation hypothesis*, in which detection performance improves with increasing number of local information signals because there is more chance that any one of the features will be detected (Kingdom, Baldwin, & Schmidtmann, 2015; Schmidtmann et al., 2015). This *probability summation* model has been further challenged recently, using radial frequency (RF) patterns as stimuli, providing evidence in favour of *additive summation* in global shape processing (Green, Dickinson, & Badcock, 2018).

This suggests that the debate around integration of local information into global percept is still open and there is no clear agreement on whether specialised global form detectors in vision exist. We point out that our experiment was not designed to provide an answer to this question. It was limited to test a well‐established regularity‐sensitive component in response to different orientation‐defined structures (i.e. Glass patterns), which share similar local stimulus statistics but elicit different global form precepts. The results we obtained were in favour of hypotheses suggesting different perceptual mechanisms for *concentric* and *radial* Glass compared with *translation* Glass patterns.

We note that the *circular* and *radial* Glass patterns generated a larger N1 than *reflection* and were the only regularities which differed from random at this latency. This was illustrated in Figure 4d,e, in which 95% CI for the difference waves of the two moirés crossed zero at approx. 170 ms, as well as in the results from mass univariate analysis (Figure 6) and topographic maps in Figure 5. Behavioural results also showed faster RTs and error rates for *circular* and *radial* compared with *reflection* patterns. Interestingly, the same results were observed in Makin et al. (2016; although the authors did not analyse the N1 component). There might be some perceptual advantage for *circular* and *radial* Glass patterns over *reflection* symmetry (which is also not captured by the predictions of the *holographic* model). The N1 component is implicated in the global structural encoding of shape information (Bentin & Golland, 2002; Doniger et al., 2001; Sehatpour, Molholm, Javitt, & Foxe, 2006). It could be that global structure in Glass patterns is extracted earlier. For example, it is suggested that *circular* and *radial* Glass patterns may be processed by fast global form detectors in dorsal visual areas, which successively feed into ventral visual areas in a heterarchical fashion (Lestou et al., 2014). This would also explain why circular and radial Glass were detected more quickly than reflection in our behavioural experiment. Alternatively, *circular* and *radial* structures may better stimulate the global form detectors in V4 (see Gallant et al., 1993; Gallant et al., 1996; Kobatake & Tanaka, 1994) than *onefold reflection* symmetry, and this could be reflected in larger and more wide‐spread negativity at the N1 latency. These are, however, speculations, which go beyond the scope of the current work and should be investigated in future research.

Finally, it is interesting that the *W*‐scores, discriminability and SPN responses to *reflection*, *circular* and *radial* Glass patterns are similar despite their disparate biological significance. Vertical *reflection* alone is a property of faces and bodies and is arguably a truthful indicator of genetic fitness (Little, Jones, & DeBruine, 2011). People and animals are attracted to *reflection* symmetry and use it in mate selection (Bertamini, Byrne, & Bennett, 2013; Little et al., 2011). However, the extrastriate visual system does not seem to treat *reflection* symmetry as special and is equally tuned to a variety of different non‐accidental relationships between elements.

## 5 CONCLUSION

The holographic weight of evidence model provides a simple and effective method to predict the perceptual goodness and neural response of several types of regularity. Our new results support the holographic model by showing similar neural response to *reflection*, *radial* and *circular* Glass patterns, but contradict the model by showing weaker responses to *translation* Glass patterns. The holographic model stresses the role of *alternation* as the perceptually relevant characteristic of moiré structures. However, it does not take into account the role of local orientations, which may be encoded by specialised form units in higher‐order visual areas.

## CONFLICT OF INTERESTS

The authors declare they have no conflicts of interest.

## Data Availability

ERP Study: Codes for experimental presentation, stimulus generation, and EEG and behavioural analysis are freely available on Open Science Framework (https://doi.org/10.17605/OSF.IO/2S36C), along with pre‐processed EEG data. Behavioural Study: Raw data and analysis code are freely available on Open Science Framework (https://doi.org/10.17605/OSF.IO/2S36C). We are happy for other researchers to analyse our data or use our codes for any purpose.

## Footnotes

^1^
We pay our gratitude and respect to Peter van der Helm for the timely and valuable influence his work has had on our research, as well as the whole vision science community.

^2^
Note that the terms *reflection*, *translation* and *rotation* are not *holographic* terms but stem from the *transformational approach* (Garner, 1974), an alternative formalisation which proposes that goodness increases with the number of structure‐preserving transformations (e.g. number of folds in reflection symmetry; number of turns in a rotation symmetry; number of repeats in repetition symmetry). In the current paper, we will refer often to *mirror*/*reflection* symmetry using the term *reflection*, as this is the term used in other SPN studies to which we have directly referred for this study (Makin et al., 2016).

^3^
The non‐significant Shapiro–Wilk tests mean we cannot confidently reject the hypothesis that the ERP data were from a normally distributed population (absence of evidence). However, it does not confirm that the data were from a normally distributed population (evidence of absence). This counterintuitive asymmetry is important when interpreting traditional *p*‐values (Masson, 2011). For further reassurance, we have conducted the same tests with non‐parametric Friedman's ANOVA. The results were very similar: that is main effect of regularity in the N1 time window, χ^2^(4) = 28.8, *p* < .001 with significant difference from *random* for *circular* and *radial* Glass (*p*s < .01); main effect for regularity in the early SPN, χ^2^(4) = 47.8, *p* < .001, with *reflection*, *circular* and *radial* Glass differing significantly from both *random* and *translation* Glass (adj. *p*s < .01); main effect of regularity in the late SPN, χ^2^(4) = 44.4, *p* < .001 with *reflection*, *circular* and *radial* Glass differing significantly from *random* (adj. *p*s < .01), *radial* − *translation* Glass being significant (adj. *p* = .01), but the *translation* − *random* being only marginal (adj. *p* = .056).
